# Supplementary material for: Sequence Order in the Range 1 to 19 by Chimpanzees on a Touchscreen Task: Processing Two-Digit Arabic Numerals
Source: Animals (Basel). 2023 Feb 21;13(5):774. doi: 10.3390/ani13050774 (PMC10000109; doi:10.3390/ani13050774)
Supplement: Supplementary file 1 [file animals-13-00774-s001.zip › Supplementary Document S5.pdf]

animals

MDPI

Supplementary material S1 (video) Ayumu VNM-Startfix 1 to 17 task (June 3, 2011).
Supplementary material S2 (video) Pal VNM-Endfix 11 to 19 task (September 13, 2013).
Supplementary material S3 (video) Pal 4 nonadjacent numerals in 1 to 19 nonmemory task (April 3, 2013).
Supplementary material S4 (video) Pal 4 nonadjacent numerals in 1 to 19 memory task (April 3, 2013).

Supplementary material S5 (document): Additional explanation of the methods and results

S5.1. Information on apparatus

The apparatus of the chimpanzee-number project in KUPRI was the computer-controlled automated system from the beginning [1,2]. It started with the "key-board plus inline-projectors" system in 1976 [3] which was replaced by the touchscreen system in 1987 [4]. The important point is that all of the number tasks are automated discrimination learning that shares many characteristics with perceptual and cognitive studies in chimpanzees and other nonhuman primates. See the review of automated methods and the technological context of chimpanzee research by Christopher Martin and Ikuma Adachi [5].

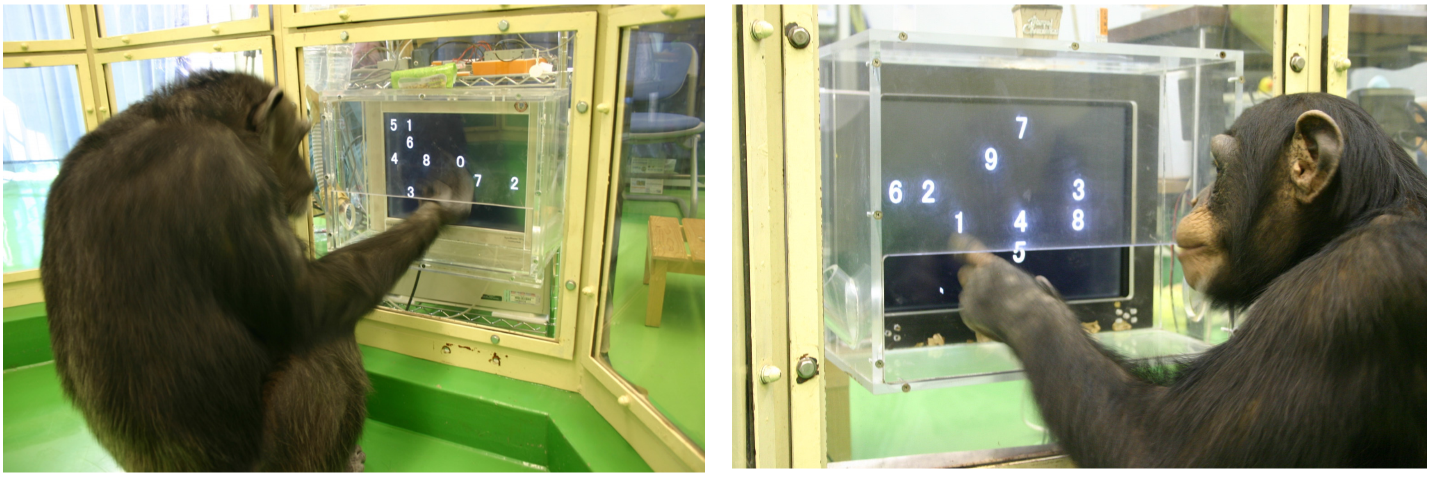

Figure S5.1. The touchscreen apparatus used by the present study. (Left): chimpanzee Ai, (Right): chimpanzee Ayumu. In the previous study before introducing 2-digit numerals, Ai learned the numerals from 0 to 9 and the other chimpanzees learned those from 1 to 9.

S5.2. Comparison of First assessment test and Second assessment test in chimpanzees

In the present study, the assessment test was conducted twice (September 2013 and March 2014). Table 4 in the Results section is based on the results of the 2nd test. Table S5-1 shows the comparison of the 1st and 2nd tests. There are four factors: As described in the Method section, four factors influence the performance of numerical ordering. First, the range of numerals was either 1 to 9 or 1 to 19. Second, the adjacency was either adjacent numerals or nonadjacent ones. Third, 'memory' means whether the task was an ordinary nonmasking task or the masking task that needed memorizing numerals. For example, suppose that five numerals such as 5-12-13-16-19 appeared on the screen. In this case, after touching the numeral 5, the other four numerals turned to become the masked pattern (black and white checker pattern). Therefore, in the masking (memory) task, the chimpanzees had to remember which numeral appeared in which position on the monitor. Fourth is the number of numerals. The present study examined the number of numerals either 3, 4, or 5. It can be predicted that the longer sequence must be more difficult than the shorter sequence in any condition. The performance in the 2nd test was slightly higher than the 1st one. According to Table S5-1, the performance increased by about 2 % in accuracy on average. The correlation between the two tests was extremely high r=0.941.

Table S5.1. Data of all six chimpanzees in the 1st and 2nd assessment test. Four factors were influencing the performance of numerical ordering. First, the range of numerals was either 1 to 9 or 1 to 19. Second, the adjacency was either adjacent or nonadjacent numerals. Third, 'memory' means whether the task was an ordinary nonmasking (nonmemory) task or the masking (memory) task that needed memorizing numerals. Fourth, the number of numerals was either 3, 4, or 5. Whatever the condition is, the chimpanzee had to touch the numerals from 1 to 9 or from 1 to 19 in ascending order. Each cell showed accuracy (% correct). Each cell is based on one test session of 50 trials.

Task				Chimpanzee Participants													
Range	Adjacency	Memory	Number of Numerals	Ai		Ayumu		Chloe		Cleo		Pan		Pal		Average	
				1st	2nd	1st	2nd	1st	2nd	1st	2nd	1st	2nd	1st	2nd	1st	2nd
1-9	Adjacent	Nonmask	3	96	98	96	98	96	94	98	98	88	90	98	98	95.3	96.0
			4	90	96	92	90	94	86	100	92	92	88	96	98	94.0	91.7
			5	90	88	90	90	84	78	86	90	88	82	96	94	89.0	87.0
		Mask	3	98	90	98	100	88	76	88	92	84	78	92	98	91.3	89.0
			4	76	74	94	88	58	60	82	88	56	42	86	90	75.3	73.7
			5	60	62	84	90	36	22	56	68	24	24	76	62	56.0	54.7
	Non-adj	Nonmask	3	100	94	100	96	96	92	94	98	94	92	98	94	97.0	94.3
			4	98	96	94	96	90	92	88	92	98	86	96	94	94.0	92.7
			5	84	92	96	94	82	92	84	78	92	88	92	90	88.3	89.0
		Mask	3	94	94	98	100	90	82	92	90	86	80	88	84	91.3	88.3
			4	90	86	92	100	68	60	76	82	48	42	80	80	75.7	75.0
			5	58	54	80	86	26	36	58	58	16	8	66	66	50.7	51.3
1-19	Adjacent	Nonmask	3	94	96	98	98	68	76	90	98	68	64	92	98	85.0	88.3
			4	92	86	96	100	54	54	80	82	48	64	98	98	78.0	80.7
			5	72	76	92	92	52	50	70	78	30	42	80	88	66.0	71.0
		Mask	3	88	88	96	98	66	46	90	94	50	80	94	92	80.7	83.0
			4	62	68	92	86	40	30	68	74	22	42	80	78	60.7	63.0
			5	32	34	62	50	8	24	44	44	8	8	60	48	35.7	34.7
	Non-adj	Nonmask	3	82	90	86	96	50	64	64	78	56	78	82	92	70.0	83.0
			4	62	72	74	86	30	62	50	54	52	58	60	70	54.7	67.0
			5	50	52	44	84	18	36	24	36	20	36	42	50	33.0	49.0
		Mask	3	86	72	64	76	66	76	58	66	60	62	74	80	68.0	72.0
			4	30	34	40	48	30	48	34	44	26	28	54	38	35.7	40.0
			5	16	14	8	8	14	18	22	16	8	16	24	26	15.3	16.3
			Average	75	75	82	85	59	61	71	75	55	57	79	79	**70.0**	**72.1**

S5.3. Comparison of chimpanzees and humans

Six human participants *partly* joined in the assessment test. Table S5-2 shows the results of all six human participants. For humans, there are three factors: they were the same in the chimpanzee test, but the fourth 'memory' factor was excluded. In chimpanzees, three factors influenced the performance of numerical ordering. First, the range of numerals was 1 to 9 or 1 to 19. Second, the adjacency was either adjacent or nonadjacent. The third was the number of numerals: either 3, 4, or 5. The longer sequence must be more difficult than the shorter sequence in any condition. The memory factor was not tested in the present study. The data shows the species difference in processing 2-digit numerals.

Table S5.2. Data of all six humans in the assessment test. Three factors were influencing the performance of numerical ordering. First, the range of numerals was either 1 to 9 or 1 to 19. Second, the adjacency was either adjacent or nonadjacent numerals. Third, the number of numerals was either 3, 4, or 5. Whatever the condition is, the human had to touch the numerals from 1 to 9 or from 1 to 19 in ascending order. Each cell showed accuracy (% correct). Each cell is based on the data of one test session that consists of 50 trials.

Task			Human Participants						
Range	Adjacency	Number of Numerals	**H1**	**H2**	**H3**	**H4**	**H5**	**H6**	**Average**
1-9	Adjacent	3	100	98	100	100	94	100	98.7
		4	100	100	98	100	98	100	99.3
		5	98	100	98	98	92	100	97.7
	Non-adj	3	100	100	100	100	98	100	99.7
		4	100	98	98	98	94	100	98.0
		5	100	96	100	98	84	92	95.0
1-19	Adjacent	3	100	96	96	98	84	98	95.3
		4	96	94	100	100	92	98	96.7
		5	100	92	88	98	92	90	93.3
	Non-adj	3	98	98	98	100	88	90	95.3
		4	94	94	100	98	94	94	95.7
		5	100	94	98	96	86	86	93.3
		Average	99	97	98	99	91	96	**96.5**

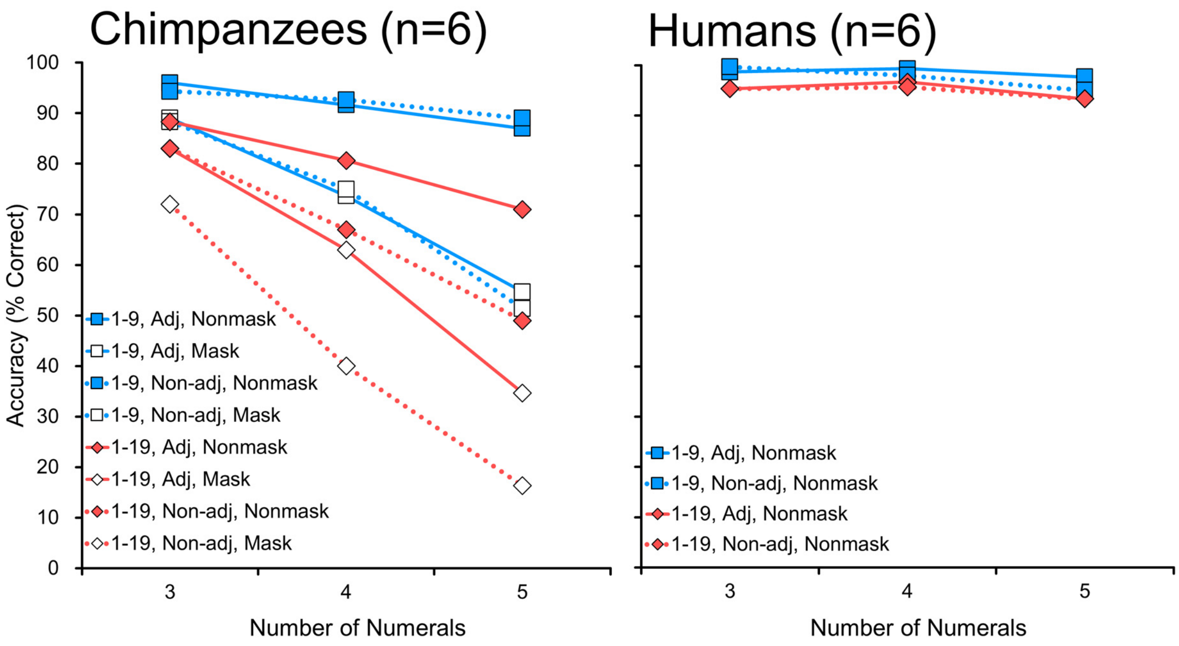

Figure S5.2. The comparison of chimpanzees (Left, n = 6) and six humans (Right, n = 6) in the combination of four factors (range, adjacency, memory, and the number of numerals). The X-axis (3, 4, and 5) showed the number of numerals presented on the screen. The Y-axis showed accuracy (% correct). The data from chimpanzees in Figure 7, Figure 8, and Figure 9 in the Results section were combined into one figure, Figure A4 in the main text. Figure A4 was again shown in the left panel of this figure for comparison with humans. For humans, the memory factor was excluded from the present test.

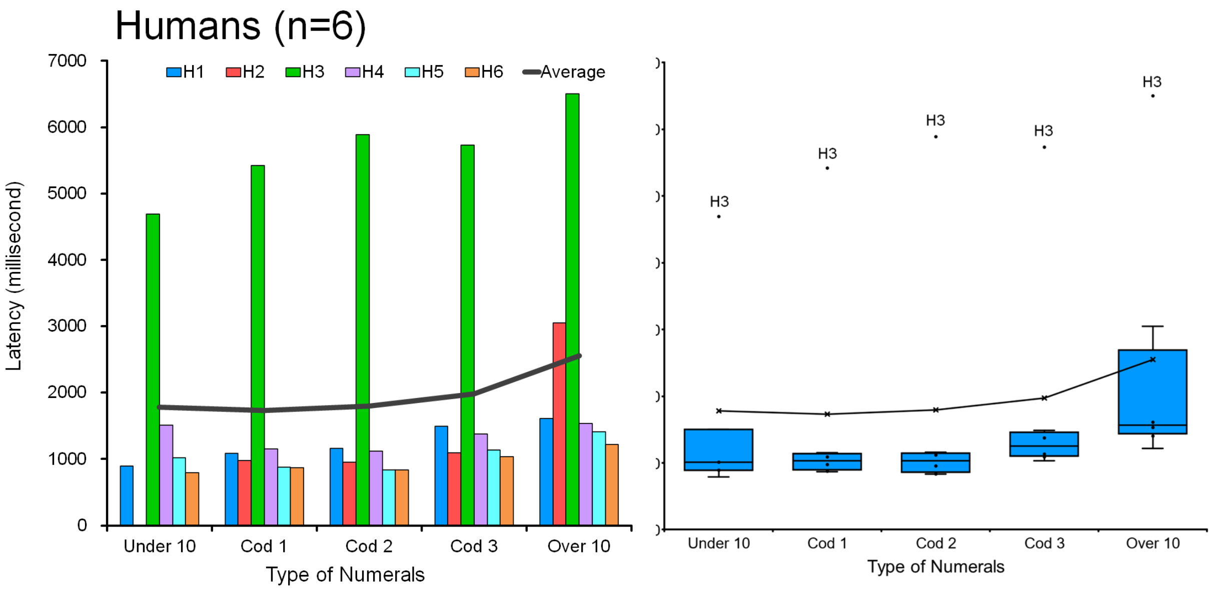

Figure S5.3. This is the response latency of touching four nonadjacent numerals in the range of 1 to 19 in humans (n = 6). (Left) This is a replication of Appendix A5. The individual data response latency (Y-axis) is plotted in the bar graph. The average performance was shown in the solid lines. The X-axis shows the five conditions of the 4 presented numerals. The condition is described in Method 3.4. Participant H3's value is out of the scale of the other five participants. (Right) The same data is plotted for showing the deviation: re-plotted in the average and SD of five other participants and the data of H3 (shown in the dots) separately. Based on this result, the data of H3 was omitted from further analysis which is shown in Figure 12 in the Results section.

References in the Supplementary material S5 (document)

1. Matsuzawa, T.; Tomonaga, M.; Tanaka, M. *Cognitive development in chimpanzees.* Springer. **2006,** https://link.springer.com/content/pdf/10.1007/4-431-30248-4.pdf
2. Matsuzawa, T. The Ai project: historical and ecological contexts. *Animal cognition,* **2003**, *6*(4), 199-211. https://doi.org/10.1007/s10071-003-0199-2
3. Asano, T.; Kojima, T.; Matsuzawa, T.; Kubota, K.; Murofushi, K. Object and color naming in chimpanzees (*Pan troglodytes*). *Proceedings of the Japan Academy. Series B, Physical and Biological Sciences,* 1982, *58*(5), 118–122.
4. Tomonaga, M.; Matsuzawa, T.; Itakura, S. Teaching ordinals to a cardinal-trained chimpanzee. *Primate Research,* **1993**, *9*(2), 67–77. (In Japanese with English summary)
5. Martin, C. F.; Adachi, I. Automated methods and the technological context of chimpanzee research. In: Hopper, L. & Ross, S. (eds) "*Chimpanzees in context*", The University of Chicago Press: Chicago, **2020,** pp. 189-207.
